# Letter from Ehrenbreitstein, Germany

**Published:** 1871-07

**Authors:** G. W. Goodner

**Affiliations:** Ehrenbreitstein, Germany


### Foreign Correspondence.

*Ehrenbreitstein, Germany, May 9th, 1871.*

Prof. N. S. Davis, M.D.—*Dear Sir,*—Perhaps it will not be entirely without interest to your readers to read a description of a few cases which have been brought into this hospital for treatment during the war just ended. As a general thing we have not had cases of great importance, but occasionally one is brought which is at least interesting physiologically. The most of the cases which I shall describe have not been in my wards during the entire treatment, and as no histories are at my disposal, shall be obliged to trust chiefly to memory. I shall therefore describe the cases as well as I can, and leave it to yourself and readers to theorize.

The first case received a ball passing in about the centre and at the junction of the neck with the shoulder of the right side, the ball being found lodged under the skin a little below and about two inches to the right of the right nipple. The external wound healed readily, but there was partial paralysis of the limbs of the left side, while the right pupil was dilated to about four times its normal size. The capillary circulation of the limbs of the left side was sluggish, the limbs being perceptibly colder than normal, while the thermometer in the left axilla showed a diminution of temperature of one degree. Professor Langenbeck examined the case, and said the ball had affected the spinal cord from concussion, and then passed through the right lung to the right side. He was troubled for a time with "night sweats," and one night I was called to him and found him senseless, throwing himself from one side of the bed to the other, and bathed in perspiration, although his temperature appeared normal and the pulse regular. The attack lasted about fifteen minutes, and was repeated once during the night, and the following morning he complained of headache and general weakness. The treatment at first was general, with no apparent effect; afterwards electricity, with daily exercise, was tried, and from that time the patient began slowly to improve. He was sent away from the hospital a few days ago, being able to walk around very well, and will probably in the course of time be able to attend to his business, if not entirely recovered.

The next case received a ball in the shoulder, just above the joint, the ball passing out near the lower angle of the scapula (right shoulder); also a ball passing through both condyles of the right femur without injury to the joint. In this case we have exemplified the tenacity of life in some cases, for we expected him to die almost every day for a month, abscesses forming in different parts of his body, bedsores on his back, and every few days hemorrhage from the wound in his shoulder. Finally the shoulder-joint became involved, and resection of the head of the humerus was performed about the middle of October, and from that time he began gaining strength, although the wound has not yet entirely healed. The wound in the knee still refuses to heal, small spiculæ of bone coming away with the pus from time to time. The surgeon says he is afraid to attempt any operation for fear he might injure the joint which is yet sound. His appetite never failed him, and he was furnished wine and beer in abundance, according to custom here. He is able to walk around, and it is hoped he will recover with the use of his knee-joint.

The next case was a lieutenant, who received a flesh wound just above the right knee, the ball passing behind the femur and injuring the peroneal nerve. There is nothing particular about this case except the pain which he always had in his foot. He had to be injected with morphia from three to five times a day, medicines being powerless. This was continued for about four months without any perceptible improvement. Finally he was ordered to be injected with water to try the mental effect. This did not relieve the pain, and he was told that morphia, when long continued, sometimes loses its effect. The injections were then stopped, and in the course of a month he was much improved, being able to sit up all day. Finally he went to Wiesbaden, to try the baths, since which time I have heard nothing from him.

There are other interesting cases, one of which I will only mention. A ball cut the skin on the top of a soldier's head without any perceptible injury to the bone. He became immediately deaf, and has remained so ever since.

A few words about Germany. When I first came here I was almost ashamed to acknowledge I was an American; now I am proud of it. As to their instruments and apparatus for dressing wounds and fractures, certainly they would lose nothing by going to America and studying the productions of "Yankee ingenuity." I have seen nothing extra which is not to be seen every day in our hospitals, and as to German education, should feel very badly to have to say it was superior to ours.

G. W. Goodner, M.D.

